# Inter-individual Differences in Heart Rate Variability Are Associated with Inter-individual Differences in Empathy and Alexithymia

**DOI:** 10.3389/fpsyg.2018.00229

**Published:** 2018-02-27

**Authors:** Alexander Lischke, Rike Pahnke, Anett Mau-Moeller, Martin Behrens, Hans J. Grabe, Harald J. Freyberger, Alfons O. Hamm, Matthias Weippert

**Affiliations:** ^1^Department of Psychology, University of Greifswald, Greifswald, Germany; ^2^Institute of Sport Science, University of Rostock, Rostock, Germany; ^3^Department of Orthopaedics, University Medicine Rostock, Rostock, Germany; ^4^Department of Psychiatry and Psychotherapy, University of Greifswald, Greifswald, Germany; ^5^HELIOS Klinikum Stralsund, Stralsund, Germany

**Keywords:** social cognition, social interaction, empathy, alexithymia, vagus nerve, high-frequency heart rate variability

## Abstract

In the present study, we investigated whether inter-individual differences in vagally mediated heart rate variability (vmHRV) would be associated with inter-individual differences in empathy and alexithymia. To this end, we determined resting state HF-HRV in 90 individuals that also completed questionnaires assessing inter-individual differences in empathy and alexithymia. Our categorical and dimensional analyses revealed that inter-individual differences in HF-HRV were differently associated with inter-individual differences in empathy and alexithymia. We found that individuals with high HF-HRV reported more empathy and less alexithymia than individuals with low HF-HRV. Moreover, we even found that an increase in HF-HRV was associated with an increase in empathy and a decrease in alexithymia across all participants. Taken together, these findings indicate that individuals with high HF-HRV are more empathetic and less alexithymic than individuals with low HF-HRV. These differences in empathy and alexithymia may explain why individuals with high HF-HRV are more successful in sharing and understanding the mental and emotional states of others than individuals with low HF-HRV.

## Introduction

Social relationships have always been of utmost importance for humans. Although the number and type of relationships may have changed over the course of evolution, the challenges and opportunities associated with social relationships may have remained the same (Dunbar, 1998). Evolutionary pressures may, thus, have selected a suite of skills that may have helped us to initiate or maintain positive relationships and to avoid or terminate negative relationships (De Waal, 2008). Of these skills, the ability to share and understand others’ emotional and mental states, which entails a simulation of these states while making a self-other distinction, appears to be of particular relevance (Preston and De Waal, 2002). Infants are already capable of sharing others’ emotional and mental states, but a full understanding of these states on basis of a self-other distinction emerges during late childhood (Frith and Frith, 2003), implying that the ability to share and understand emotional and mental states comprise various processes that become more and more complex throughout our development. It is important to note that these processes involve a simulation of others’ emotional and mental states on the neural and autonomic level, an interpretation of the simulated states on basis of the corresponding neural and autonomic changes, and a distinction between the simulated and observed states (Preston and De Waal, 2002; De Waal, 2008). These processes have been linked to inter-individual differences in empathy, a personality trait describing an individual’s awareness of *other’s* emotional and mental states (Deutsch and Madle, 1975), and alexithymia, a personality trait describing an individual’s awareness of *one’s own* emotional and mental states (Nemiah et al., 1976). Individuals with low levels of empathy and/or high levels of alexithymia are severely impaired in their ability to share and understand emotional and mental states of others and the self (e.g., Baron-Cohen et al., 1999; Parker et al., 2001; Dolan and Fullam, 2004; Moriguchi et al., 2007; Silani et al., 2008; Decety et al., 2013), which may explain why these individuals frequently have difficulties to establish and maintain positive relationships (e.g., Rilling et al., 2007; Chiu et al., 2008; Mokros et al., 2008; Feldmanhall et al., 2013). It is, thus, not surprising that the interest for biomarkers indicating such impairments has steadily been growing over the last decade. It should be noted, however, that the search for these biomarkers is more complex than initially thought (Kapur et al., 2012; Davis et al., 2015).

Vagally mediated heart rate variability (vmHRV), an index of parasympathetically induced changes in consecutive heart beats (Berntson et al., 1997), has been suggested to be a promising biomarker for inter-individual differences in social behavior and social cognition (Porges, 2007; Thayer and Lane, 2009). Inter-individual differences in vmHRV reflect inter-individual differences regarding the engagement of prefrontal and (para-)limbic brain regions during the regulation of emotional and cognitive processes (Porges, 2007; Thayer and Lane, 2009; Thayer et al., 2012), indicating that inter-individual differences in vmHRV may work as biomarker for inter-individual differences in the social domain. Individuals with high vmHRV are more efficient in establishing and maintaining positive relationships than individuals with low vmHRV (e.g., Kogan et al., 2014; Beffara et al., 2016; Lischke et al., 2018), implying that the relationships of individuals with high vmHRV are more characterized by mutual understanding than the relationships of individuals with low vmHRV (Kok and Fredrickson, 2010). Inter-individual differences regarding the ability to share and understand emotional and mental states may explain why individuals with high vmHRV are more likely to achieve a mutual understanding in social relationships than individuals with low vmHRV. Individuals with high vmHRV may be more efficient in regulating emotional and cognitive processes during the simulation of the respective states (e.g., Geisler et al., 2010, 2013; Williams et al., 2015) and may be more efficient in regulating cognitive processes that are necessary for the interpretation of the respective states (e.g., Hansen et al., 2003; Segerstrom and Nes, 2007; Luft et al., 2009) than individuals with low vmHRV, which may result in a more efficient sharing and understanding of the respective states in individuals with high as compared to low vmHRV (e.g., Cote et al., 2011; Quintana et al., 2012a; Lischke et al., 2017). However, individuals with high and low vmHRV do not only differ from one another with respect to processes that are relevant for the sharing and understanding of emotional and mental states, but also with respect to personality traits that are relevant for the sharing and understanding of these states. Empathy related personality traits, like, for example, compassion for other’s emotional and mental states, are more pronounced in individuals with high than low vmHRV (e.g., Oveis et al., 2009; Kogan et al., 2014; Stellar et al., 2015), whereas alexithymia related personality traits, like, for example, difficulties in identifying or describing one’s own emotional and mental states, appear to be more pronounced in individuals with low than high vmHRV (e.g., Fukunishi et al., 1999; Panayiotou and Constantinou, 2017). It should be noted, however, that the association between inter-individualdifferences in vmHRV and inter-individual differences in alexithymia related personality traits is less clear than the association between inter-individual differences in vmHRV and inter-individual differences in empathy related personality traits (e.g., Virtanen et al., 2003; Zohar et al., 2013), indicating a need for further studies investigating this association. Similarly, there is a need to further study the association between inter-individual differences in vmHRV and inter-individual differences in empathy related personality traits because this association has only been investigated in a few studies (e.g., Oveis et al., 2009; Kogan et al., 2014; Stellar et al., 2015).

In the present study, we addressed these issues in a relatively large and homogenous sample of healthy participants by measuring inter-individual differences in vmHRV as well as inter-individual differences in empathy and alexithymia related personality traits. On basis of previous studies (e.g., Fukunishi et al., 1999; Oveis et al., 2009; Kogan et al., 2014; Stellar et al., 2015; Panayiotou and Constantinou, 2017), we expected participants with high vmHRV to report more empathy and less alexithymia than participants with low vmHRV. We also expected inter-individual differences in vmHRV to be differently associated with inter-individual differences in empathy and alexithymia across all participants.

## Materials and Methods

### Participants

According to an *a priori* power analysis with G^∗^Power3 (Faul et al., 2007), we had to recruit 90 participants to be able to detect medium effect sizes in our categorical (*f* = 0.30, 1-β = 80, α = 0.05) and dimensional (*f*^2^= 0.15, 1-β = 80, α = 0.05) analyses regarding the association between inter-individual differences in vmHRV and inter-individual differences in empathy or alexithymia. In order to be considered for recruitment, participants had to pass a screening concerning the presence of current mental disorders and the use of current psychotropic medication. Female participants were not considered for recruitment to control sex-differences in empathy (Christov-Moore et al., 2014), alexithymia (Levant et al., 2009) and vmHRV (Koenig and Thayer, 2016). We, thus, recruited 90 male participants at the Institute of Sport Science of the University of Rostock (see **Table 1**). All participants provided written-informed consent to the study protocol that was approved by the ethics committee of the University of Rostock and carried out in accordance with the Declaration of Helsinki.

**Table 1:** Participant characteristics.

	*M*	*SEM*
Age (years)	26.20	0.43
Body mass index (kg/m^2^)	24.05	0.27
Physical activity (h/w)	7.05	3.70
Respiratory activity (Log-pHF-HRV, Hz)	-0.72	0.01
Heart rate variability (Log HF-HRV, ms^2^)	2.69	0.05
Empathy (EQ-15)^a^	16.42	0.01
Alexithymia (TAS-20)^b^	44.13	1.15

*Log-pHF-HRV = log-transformed peak of high frequency heart rate variability, Log-HF-HRV = log-transformed high frequency heart rate variability, EQ-15 = Empathy Quotient 15 (Allison et al., 2011), TAS-20 = Toronto Alexithymia Scale 20 (Bagby et al., 1994a,b).*

*^a^Data was available for 79 participants.*

*^b^Data was available for 89 participants.*

### Procedure

After arriving at the laboratory, participants were asked to use the bathroom to control for the effects of bladder filling and gastric distension on vmHRV (Quintana and Heathers, 2014). Participants were then seated in a comfortable chair and prepared for a 5 min heart rate (HR) recording. As recently recommended (Quintana et al., 2016), participants were instructed to breathe spontaneously and to keep their eyes open during the recording. After the recording, participants completed questionnaires assessing inter-individual differences in empathy (Allison et al., 2011) and alexithymia (Bagby et al., 1994a,b).

### Heart Rate Variability

HR was recorded continuously with a chest belt system, the RS800 HR monitor (Polar Electro Oy, Kempele, Finland), providing a sampling rate of 1000 Hz. HR monitors like the RS800 have been shown to record changes in consecutive heart beats as accurate as conventional electrocardiograms (Weippert et al., 2010; Quintana et al., 2012b), indicating that the recorded data were valid and reliable measures of instantaneous HR. Device specific software (Polar ProTrainer 5; Polar Electro Oy, Kempele, Finland) was used to transfer the recorded data to a computer for further data processing with Kubios HRV 2.2 (Tarvainen et al., 2014). Following established guidelines (Task Force of the European Society of Cardiology, 1996), the recorded data was visually inspected, detrended (smoothn priors: λ = 500) and, whenever necessary, corrected using adaptive filtering. Thereafter, the recorded data was subjected to a spectral analysis to determine HF-HRV, a measure of vagally mediated cardiac activity (Berntson et al., 1997), and the peak of HF-HRV (pHF-HRV), a measure of respiratory activity (Berntson et al., 1997). Besides these measures, no further measures were determined to avoid interpretational issues arising from the use of measures that do not clearly reflect vagally mediated cardiac activity (Berntson et al., 1997).

### Questionnaires

The Empathy Quotient (EQ-15; Allison et al., 2011) is a 15 item self-report questionnaire for the assessment of empathy. The EQ-15 comprises a main scale for the assessment of global empathy and several subscales for the assessment of specific aspects of empathy (e.g., emotional reactivity or social skills). However, the subscales are highly inter-correlated with one another (Muncer and Ling, 2006; Allison et al., 2011), implying that the EQ-15 measures empathy as an unidimensional rather than multidimensional construct. Following previous recommendations (Muncer and Ling, 2006; Allison et al., 2011), we only considered the main scale in our analyses. The main scale had good psychometric properties [α = 0.75], which were comparable to those that have previously been reported (Muncer and Ling, 2006; Allison et al., 2011)

The Toronto Alexithymia Scale 20 (TAS-20; Bagby et al., 1994a,b) is a 20 item self-report questionnaire for the assessment of alexithymia. The TAS-20 consists of a main scale assessing global differences in alexithymia and of several subscales assessing specific differences in alexithymia (e.g., difficulties in identifying or describing feelings). However, the high correlations between the different subscales and the low reliabilities of some subscales complicate the interpretation of the respective subscales (Kooiman et al., 2002; Muller et al., 2003). Accordingly, it has been suggested that the TAS-20 may be better suited to measure alexithymia as an unidimensional rather than multidimensional construct (Vorst and Bermond, 2001). We, therefore, considered the main scale but not the subscales in our analyses. The main scale had excellent psychometric properties [α = 0.86], which were similar to those that have previously been reported (Bagby et al., 1994a,b).

### Statistical Analysis

All statistical analyses were conducted with SPSS 22 (SPSS Inc., Chicago, IL, United States). To investigate whether inter-individual differences in vmHRV would be associated with inter-individual differences in empathy and alexithymia, dimensional and categorical analyses were performed. In all analyses, pre-cautions were taken to control for inter-individual differences in age (years), body mass index (BMI, kg/m^2^), physical activity (h/w) and respiratory activity (pHF-HRV, Hz) that may contribute to inter-individual differences in vmHRV (Quintana et al., 2016). Inter-individual differences in physical and respiratory activity were of particular concern because participants were recruited at a Sport Science facility, where the prevalence of athletes that differ from non-athletes in vmHRV due to inter-individual differences in physical and respiratory activity is higher than in the general population (Aubert et al., 2003). For the categorical analyses, analyses of covariance (ANCOVAs) were computed to determine whether participants with high and low HF-HRV would show inter-individual differences in empathy and alexithymia. Assignment of participants to the high and low HF-HRV group was based on a median split. For the dimensional analyses, multiple hierarchical regression analyses were computed to determine whether inter-individual differences in HF-HRV would be differentially associated with inter-individual differences in empathy and alexithymia across all participants. Prior to all analyses, HF-HRV and pHF-HRV were log transformed (log 10) to account for deviations from normality distribution. The significance level for the analyses was set at *p* ≤ 0.05 (two-tailed). In addition to the significance level (*p*), effect sizes ($\eta_{p}^{2}$ and *R^2^*) were determined to facilitate the interpretation of significant findings (Cohen, 1988).

## Results

### Inter-individual Differences in Heart Rate Variability and Inter-individual Differences in Empathy

**FIGURE 1:**
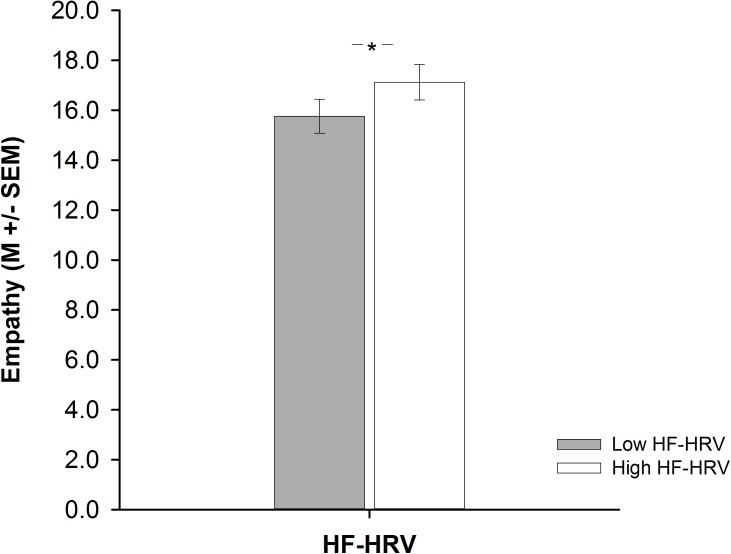
Barplots demonstrating differences in empathy between participants with high (white bars) and low (gray bars) high-frequency heart rate variability (HF-HRV). Bars represent M ± SEM. ^∗^*p* ≤ 0.05.

A one-way ANCOVA showed that participants with high HF-HRV reported more empathy than participants with low HF-HRV [*F*(1,73) = 6.51, *p* = 0.013, $\eta_{p}^{2}$ = 0.08; see **Figure 1**], independent of inter-individual differences in age, BMI, physical or respiratory activity. Across all participants, inter-individual differences in HF-HRV were positively associated with inter-individual differences in empathy as indicated by a multiple regression analysis [*t*(73) = 2.17, *p* = 0.033; see **Table 2**]. The multiple regression analysis also indicated that there was no association of inter-individual differences in age, BMI, physical or respiratory activity with inter-individual differences in empathy [all *p* > 0.339; see **Table 2**]. Most of the variance regarding inter-individual differences in empathy was, thus, explained by inter-individual differences in HF-HRV [*F*(1,73) = 4.71, *p* = 0.033; see **Table 2**], not by inter-individual differences in age, BMI, physical or respiratory activity [*F*(4,74) = 0.19, *p* = 0.944; see **Table 2**]. More precisely, inter-individual differences in HF-HRV explained 6% of variance regarding inter-individual differences in empathy in addition to the 2% of variance that tended to be explained by inter-individual differences in age, BMI, physical or respiratory activity.

**Table 2:** Association between inter-individual differences in heart rate variability and inter-individual differences in empathy across all participants.

	Empathy (EQ-15^a^)		
Predictors	*R^2^*	*ΔR^2^*	β
Step 1	0.01	0.01	
Age (years)			-0.08
Body mass index (kg/m^2^)			0.67
Physical activity (h/w)			0.00
Respiratory activity (Log-pHF-HRV, Hz)			0.01
Step 2	0.07	0.06^∗^	
Age (years)			-0.09
Body mass index (kg/m^2^)			0.11
Physical activity (h/w)			-0.06
Respiratory activity (Log-pHF-HRV, Hz)			-0.05
Heart rate variability (Log-HF-HRV, ms^2^)			0.26^∗^

*Log-pHF-HRV = log-transformed peak of high frequency heart rate variability, Log-HF-HRV = log transformed high frequency heart rate variability, EQ-15 = Empathy Quotient 15 (Allison et al., 2011).*

*^a^Data was available for 79 participants.*

*^∗^p ≤ 0.05.*

### Inter-individual Differences in Heart Rate Variability and Inter-individual Differences in Alexithymia

**FIGURE 2:**
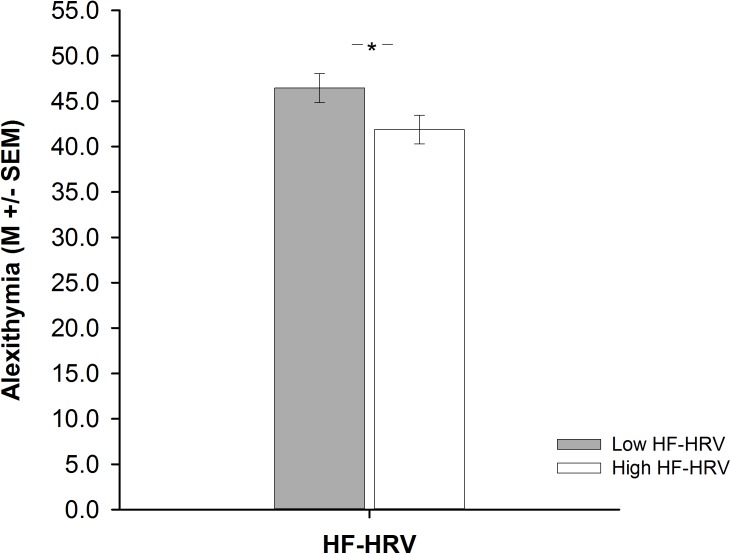
Barplots demonstrating differences in alexithymia between participants with high (white bars) and low (gray bars) high-frequency heart rate variability (HF-HRV). Bars represent M ± SEM. ^∗^*p* ≤ 0.05.

A one-way ANCOVA revealed that participants with high HF-HRV reported less alexithymia than participants with low HF-HRV [*F*(1,83) = 3.99, *p* = 0.049, $\eta_{p}^{2}$ = 0.05, see **Figure 2**], irrespective of inter-individual differences in age, BMI, physical or respiratory activity. Across all participants, inter-individual differences in HF-HRV were negatively associated with inter-individual differences in alexithymia as indicated by a multiple regression analysis [*t*(83) = -2.02, *p* = 0.047; see **Table 3**]. The multiple regression analysis further indicated that inter-individual differences in age were also negatively associated with inter-individual differences in alexithymia [*t*(83) = -2.81, *p* = 0.006; see **Table 3**] and that inter-individual differences in BMI, physical or respiratory activity were not associated with inter-individual differences in alexithymia [all *p* > 0.416; see **Table 3**]. However, inter-individual differences in alexithymia were more relevant for explaining inter-individual differences in HF-HRV [*F*(1,83) = 4.07, *p* = 0.047; see **Table 3**] than inter-individual differences in age, BMI, physical or respiratory activity [*F*(4,84) = 1.98, *p* = 0.106; see **Table 3**]. Inter-individual differences in HF-HRV explained 4% of variance regarding inter-individual differences in alexithymia in addition to the 9% of variance that tended to be explained by inter-individual differences in age, BMI, physical or respiratory activity.

**Table 3:** Association between inter-individual differences in heart rate variability and inter-individual differences in alexithymia across all participants.

	Alexithymia (TAS-20^a^)		
Predictors	*R^2^*	*ΔR^2^*	β
Step 1	0.09	0.09	
Age (years)			-0.29
Body mass index (kg/m^2^)			0.08
Physical activity (h/w)			0.04
Respiratory activity (Log-pHF-HRV, Hz)			0.10
Step 2	0.13	0.04^∗^	
Age (years)			-0.29^∗∗^
Body mass index (kg/m^2^)			0.04
Physical activity (h/w)			0.09
Respiratory activity (Log-pHF-HRV, Hz)			0.60
Heart rate variability (Log-HF-HRV, ms^2^)			-0.22^∗^

*Log-pHF-HRV = log-transformed peak of high frequency heart rate variability, Log-HF-HRV = log transformed high frequency heart rate variability, TAS-20 = Toronto Alexithymia Scale 20 (Bagby et al., 1994a,b).*

*^a^Data was available for 89 participants.*

*^∗^p ≤ 0.05, ^∗∗^p < 0.01.*

## Discussion

In the present study, we investigated the association of inter-individual differences in vmHRV with inter-individual differences in empathy and alexithymia related personality traits. Inter-individual differences in vmHRV were determined on basis of inter-individual differences in resting state HF-HRV and inter-individual differences in empathy and alexithymia were determined on basis of inter-individual differences in questionnaire scores. In line with our expectations, we found a positive association between inter-individual differences in vmHRV and inter-individual differences in empathy. Our categorical analyses revealed that participants with high vmHRV were more empathetic than participants with low vmHRV and our dimensional analyses indicated that an increase in vmHRV was associated with an increase in empathy across all participants. Also as expected, we found a negative association between inter-individual differences in vmHRV and inter-individual differences in alexithymia. Our categorical analyses showed that participants with high vmHRV were less alexithymic than participants with low vmHRV and our dimensional analyses indicated that an increase in vmHRV was associated with a decrease in alexithymia across all participants.

Previous studies revealed a similar association of inter-individual differences in vmHRV with inter-individual differences in empathy and alexithymia related personality traits (e.g., Fukunishi et al., 1999; Oveis et al., 2009; Kogan et al., 2014; Stellar et al., 2015; Panayiotou and Constantinou, 2017). With respect to empathy it is noteworthy that individuals with high vmHRV show more agreeableness with others and more compassion for others’ emotional or mental states than individuals with low vmHRV (e.g., Oveis et al., 2009; Kogan et al., 2014; Stellar et al., 2015). Agreeableness is a personality trait that is closely related to compassion and compassion is a personality trait that is closely related to empathetic concern (Goetz et al., 2010), a distinct dimension of empathy that has been regarded as an important precursor of prosocial behavior (Batson and Shaw, 1991). Inter-individual differences in vmHRV may, thus, be differentially associated with distinct empathy dimensions, implying the possibility of positive associations with empathy dimensions that facilitate prosocial behavior, such as empathetic concern (e.g., Toi and Batson, 1982; Batson et al., 1983), and negative associations with empathy dimensions that impair prosocial behavior, such as empathetic distress (e.g., Toi and Batson, 1982; Batson et al., 1983). In the present study, we were unable to test this possibility because the psychometric properties of our empathy questionnaire argued against the use of the questionnaire’s subscales in the respective analyses. Future studies should, thus, employ empathy questionnaires with psychometrically sound subscales to further elucidate the association between inter-individual differences in vmHRV and inter-individual differences in empathy. With respect to alexithymia it is noteworthy that individuals with high vmHRV report fewer difficulties in identifying or describing their own emotional and mental states than individuals with low vmHRV (e.g., Fukunishi et al., 1999; Panayiotou and Constantinou, 2017). However, the association between inter-individual differences in vmHRV and inter-individual differences in alexithymia seem to be more pronounced among younger (e.g., Fukunishi et al., 1999; Panayiotou and Constantinou, 2017) than older (e.g., Virtanen et al., 2003; Zohar et al., 2013) individuals. Future studies should, therefore, investigate this association among individuals showing a wider age range than those individuals that have been included in the present study. These studies should also employ alexithymia questionnaires with psychometrically sound subscales to explore whether inter-individual differences in vmHRV are differentially associated with distinct dimensions of alexithymia as suggested by previous studies (e.g., Fukunishi et al., 1999; Panayiotou and Constantinou, 2017). In the present study, we were unable to perform the respective analyses because of the problematic subscale structure of our alexithymia questionnaire. Taken together, the findings of the present and previous studies suggest that inter-individual differences in vmHRV are associated with inter-individual differences regarding the ability to share and understand emotional and mental states of others and the self.

Assuming an association of inter-individual differences in vmHRV with inter-individual differences in empathy and alexithymia may help to explain why individuals with high vmHRV are more successful in establishing and maintaining positive relationships than individuals with low vmHRV (e.g., Kok and Fredrickson, 2010; Beffara et al., 2016; Lischke et al., 2018). Individuals with high vmHRV may be more efficient in simulating and interpreting emotional and mental states under a self-other awareness than individuals with low vmHRV, which may increase the likelihood of mutual understanding that is necessary for the establishment and maintenance of positive relationships. Inter-individual differences regarding the regulation of cognitive and emotional processes that are relevant for the simulation and interpretation of emotional and mental states, like, for example, the control of emotions (e.g., Geisler et al., 2010, 2013; Williams et al., 2015) or the allocation of attention (e.g., Hansen et al., 2003; Segerstrom and Nes, 2007; Luft et al., 2009), may contribute to these differences. In this respect it is noteworthy that individuals with high vmHRV outperform individuals with low vmHRV on tasks that require the inference of others’ states on basis of facial and/or vocal cues (e.g., Cote et al., 2011; Quintana et al., 2012a; Lischke et al., 2017), indicating the plausibility of the aforementioned assumptions.

With respect to the neurobiological mechanisms mediating the association of inter-individual differences in vmHRV with inter-individual differences in empathy and alexithymia, it is important to note that a similar set of prefrontal and (para-)limbic brain regions is engaged during the simulation and interpretation of emotional and mental states as during the regulation of cardiac activity (Bernhardt and Singer, 2012; Thayer et al., 2012; Wingbermuhle et al., 2012). Of these brain regions, the anterior cingulate cortex, the insula and amygdala are of particular relevance because functional and structural changes in these brain regions are associated with changes in empathy and alexithymia (e.g., Singer et al., 2004; Moriguchi et al., 2007; Reker et al., 2010; Banissy et al., 2012; Klimecki et al., 2012; Bernhardt et al., 2014; Grabe et al., 2014; Goerlich-Dobre et al., 2015) as well as with changes in vmHRV (e.g., Gianaros et al., 2004; Lane et al., 2009; Sakaki et al., 2016; Winkelmann et al., 2017). Following previous suggestions that changes in vmHRV serve as a proxy for changes in prefrontal activity and prefrontal-(para-)limbic connectivity (Porges, 2007; Thayer and Lane, 2009; Thayer et al., 2012), we assume that inter-individual differences in vmHRV reflect inter-individual differences in empathy and alexithymia that are due to inter-individual differences in prefrontal activity and prefrontal-(para-)limbic connectivity. More precisely, we assume that individuals with high vmHRV are more empathetic and less alexithymic than individual with low vmHRV because individuals with high vmHRV are more efficient in recruiting prefrontal and (para-)limbic brain regions implicated in the simulation and interpretation of emotional and mental states than individuals with low vmHRV. In this respect it is noteworthy that individuals with autism, a disorder that is characterized by alterations in empathy and alexithymia (Hill et al., 2004; Dziobek et al., 2008), show alterations in a prefrontal and (para-)limbic brain regions (e.g., Baron-Cohen et al., 1999; Dziobek et al., 2006; Silani et al., 2008; Wicker et al., 2008; Ecker et al., 2012) as well as alterations in vmHRV (e.g., Mathewson et al., 2011; Kuiper et al., 2017). Inter-individual differences in vmHRV may, thus, indicate inter-individual differences regarding the recruitment of prefrontal and (para-)limbic brain regions during the simulation and interpretation of emotional and mental states in healthy as well as in mentally disordered individuals, implying that inter-individual differences in vmHRV may indeed serve as biomarker for inter-individual differences in empathy and alexithymia.

The findings of the present study suggest that inter-individual differences in vmHRV are associated with inter-individual differences in empathy and alexithymia, presumably because of inter-individual differences in prefrontal activity and prefrontal-(para-)limbic connectivity during the simulation and interpretation of emotional and mental states. The present findings are not only consistent with findings of previous studies revealing an association between inter-individual differences in vmHRV and inter-individual differences in the regulation of emotional and cognitive processes that are necessary for the simulation and interpretation of emotional and mental states (e.g., Hansen et al., 2003; Segerstrom and Nes, 2007; Luft et al., 2009; Geisler et al., 2010; Geisler et al., 2013; Williams et al., 2015), but also with findings of previous studies suggesting an association between inter-individual differences in vmHRV and inter-individual differences in prefrontal activity and prefrontal-(para-)limbic connectivity during the regulation of emotional and cognitive processes that are necessary for the simulation and interpretation of emotional and mental states (e.g., Gianaros et al., 2004; Lane et al., 2009; Sakaki et al., 2016). Taken together, these findings corroborate our assumption that inter-individual differences in vmHRV are associated with inter-individual differences regarding the ability to share and understand emotional and mental states of others and the self. However, whether inter-individual differences in vmHRV really have the potential to work as a biomarker for inter-individual differences in empathy and alexithymia remains to be determined in future studies that are explicitly designed for these types of investigations (Kapur et al., 2012; Davis et al., 2015). These studies should employ correlational and experimental study designs in a cross-sectional or longitudinal way to investigate the aforementioned associations on the behavioral and neural level in healthy and mentally disordered individuals with performance and questionnaire based measures of empathy, alexithymia and social behavior. Otherwise it will be difficult to determine whether inter-individual differences in vmHRV qualify as a biomarker for inter-individual differences in empathy and alexithymia.

## Author Contributions

AL, AM-M, and RP designed the study. AM-M and MW collected the data. AL and RP analyzed the data. AL wrote the manuscript. AH, AM-M, HF, HG, MB, MW, and RP contributed to writing, reviewing and editing of the manuscript. All authors approved the final version of the manuscript.

## Disclosure

Funding for this study was supported by an Open Access Publishing grant that was provided by the German Research Foundation (DFG) and the University of Rostock. AL was supported by a grant provided by the German Research Foundation (DFG; LI 2517/2-1). The funding source had no further role in study design, in the collection, analysis and interpretation of data; in the writing of the report; and in the decision to submit the paper for publication.

## Conflict of Interest Statement

The authors declare that the research was conducted in the absence of any commercial or financial relationships that could be construed as a potential conflict of interest.
